# Sensitivity and Precision of Search Strategies Built Using a Text‐Mining Word Frequency Tool (PubReMiner) Compared to Current Best Practice for Building Search Strategies: A Study Within a Review (SWAR)

**DOI:** 10.1002/cesm.70074

**Published:** 2026-02-18

**Authors:** Andrew Dullea, Marie Carrigan, Lydia O'Sullivan, Isabelle Delaunois, Helen Clark, Martin Boudou, Martina Giusti, Kieran A. Walsh, Patricia Harrington, Susan M. Smith, Máirín Ryan

**Affiliations:** ^1^ Discipline of Public Health & Primary Care, School of Medicine, Trinity College Dublin, The University of Dublin Dublin Co. Dublin Ireland; ^2^ Health Technology Assessment Directorate, Health Information and Quality Authority Mahon Co. Cork Ireland; ^3^ Health Research Board-Trials Methodology Research Network, College of Medicine, Nursing and Health Sciences National University of Galway Galway Co. Galway Ireland; ^4^ European Food Safety Authority Parma Italy; ^5^ HSE Health Library Ireland, Sligo University Hospital Sligo Co. Sligo Ireland; ^6^ Department of Experimental and Clinical Medicine University of Florence (Università degli Studi di Firenze) Florence Tuscany Italy; ^7^ School of Pharmacy, University College Cork Cork Co. Cork Ireland; ^8^ Department of Pharmacology and Therapeutics Trinity College Dublin, The University of Dublin Dublin Co. Dublin Ireland

**Keywords:** information retrieval, study within a review, SWAR, systematic search methods, text‐mining

## Abstract

**Objective:**

PubReMiner is a text‐mining tool that analyses a seed set of citations to assess word frequency in titles, abstracts, and Medical Subject Headings (MeSH). This study aimed to determine the sensitivity and precision of search strategies developed using the PubReMiner tool compared to conventional search strategies developed by a librarian at our institution.

**Methods:**

Twelve consecutive reviews conducted at our center were included from September 2023 to January 2025. These reviews included various types of evidence synthesis, including rapid reviews and systematic reviews, covering a variety of topics. One librarian developed a comprehensive search strategy, which included a conventional MEDLINE search for each review. Separately, two librarians independently developed MEDLINE search strategies using PubReMiner‐generated word frequency tables (PubReMiner 1 and PubReMiner 2). All search strategies were constructed by experienced librarians using predefined work instructions. Primary outcomes were sensitivity and precision. Secondary outcomes included the number needed to read, the number of unique references retrieved, and the time taken to construct each strategy.

**Results:**

Sensitivity of PubReMiner strategies was generally lower than that of conventional strategies; however, in one review, PubReMiner achieved a higher sensitivity (83.87%) than the conventional strategy (58.06%). Only the sensitivity outcome showed a statistically significant difference between search methods (Friedman test *p* = 0.0065). No statistically significant difference in precision between the searches was identified. PubReMiner strategies were typically faster to construct but yielded inconsistent performance across reviews and between librarians.

**Conclusion:**

While PubReMiner offers efficiency advantages, its inconsistent performance in retrieving relevant studies suggests that it should not replace conventional search strategies. The study illustrates the value of multi‐review SWARs in producing evidence that informs evidence synthesis practices.

## 1 Introduction

Evidence syntheses, such as systematic reviews and rapid reviews, inform evidence‐based decision‐making in healthcare and health policy. In order to conduct robust evidence synthesis, information must be synthesised in a systematic manner, and an accurate and comprehensive search strategy is critical to achieve this. As new and more complex methods and tools for evidence synthesis emerge, they are accompanied by a growing uncertainty and lack of consensus regarding how best to implement these innovations in practice. A Study Within A Review (SWAR) is a form of embedded methodological research that can be used to address such uncertainties. SWARs are typically embedded within a systematic review or other evidence syntheses in order to generate evidence and inform decisions about how we plan, do, or share the findings of future systematic reviews or other evidence syntheses [1].

While a number of SWARs have been registered in the SWAR repository hosted by The Northern Ireland Network for Trials Methodology Research to date, there is a notable absence of SWARs embedded across multiple reviews. This issue has also been highlighted by other researchers [1, 2]. Embedding a SWAR across diverse evidence syntheses may enhance the generalisability and external validity of its findings, an especially relevant approach when the effectiveness of a method is likely to vary between reviews. This approach could be taken to evaluate any aspect of the evidence synthesis process, one area in need of robust evaluation is the development of search strategies using automation and artificial intelligence (AI) tools.

In recent years, a range of automated and AI‐driven tools have become available, offering new ways to search for relevant literature. Such tools include ResearchRabbit, Semantic Scholar, PubReMiner and large language models (LLMs) such as ChatGPT [3, 4, 5, 6, 7]. Despite growing interest, these tools require evaluation before they can be recommended for routine use in research workflows.

PubReMiner is a text‐mining tool that can be used to identify frequently used words from key publications and can be used to help efficiently build search strategies. PubReMiner works with MEDLINE to determine the frequency of free‐text and Medical Subject Headings (MeSH) terms used in key publications by inputting their PubMed Identifiers (PMIDs). This differs from conventional search strategy building techniques, where the researcher is required to identify the MeSH terms, keywords and relevant synonyms themselves [8]. Such automation methods may be attractive for researchers, particularly healthcare professionals, who may not have comprehensive skills in search strategy development, or may not have access to expertise from an information specialist or librarian [9]. However, the use of such a tool in this way would only be of use if acceptable sensitivity and precision were demonstrated.

We embedded a SWAR across multiple reviews conducted by the Health Technology Assessment (HTA) directorate in the Health Information and Quality Authority (HIQA), Ireland, over a 14‐month period. In this study, we aimed to determine the sensitivity and precision of MEDLINE searches developed solely using high‐frequency words/MeSH terms generated by PubReMiner compared to conventional peer‐reviewed MEDLINE searches.

## 2 Materials and Methods

This study was prospectively registered on the SWAR Repository Store and reviews were consecutively recruited between September 2023 and January 2025 at HIQA's HTA directorate [10]. PMIDs required for the PubReMiner tool to produce word frequency tables were obtained from topic exploration or informal scoping performed by researchers working on reviews recruited into the SWAR.

Review protocols and a search builder form with pre‐specified concepts for each review were made available to all librarians. Librarians were given the opportunity to pose questions about the reviews to the SWAR project lead (AD), and, if needed, the individual review lead authors. This was done to ensure clarity in relation to the topic under assessment for the librarians external to HIQA who were conducting the PubReMiner searches, as such, generally less familiar with the topics. The librarians conducting the conventional and PubReMiner searches (MC, ID, HC) each had over 20 years of experience conducting systematic searches.

### 2.1 Conventional MEDLINE Searches

A comprehensive peer‐reviewed search strategy was developed in line with in‐house processes for each review by an experienced librarian based in the HTA directorate. These searches were externally peer‐reviewed by a Health Service Executive (HSE) Evidence Team Librarian with no affiliation to this SWAR using the Peer‐Review of Electronic Search Strategies (PRESS) checklist [11]. The portion of this search carried out on MEDLINE via EBSCOhost was considered the ‘conventional MEDLINE search’. For the purposes of this study, a work instruction was developed *a priori* to capture this established pre‐existing process.

### 2.2 PubReMiner Searches

Two experienced librarians who were external to the HTA directorate independently conducted PubReMiner searches. All PubReMiner searches were conducted via EBSCOhost within 1 week of the conventional MEDLINE search, where possible to mitigate against any biases introduced by the date the searches were conducted.

Before recruiting reviews into the SWAR, we drafted a work instruction for developing search strategies using the PubReMiner tool (see Supporting Materials). The aim of this work instruction was to ensure consistent practices in the use of the PubReMiner tool and to minimise variation between librarians (i.e., inter‐operator variability). We applied this draft work instruction to a pilot review and compared how the searches performed. Following a de‐brief meeting where feedback and comments were sought, we updated the work instruction and applied it to a second pilot review. Following observed improvements in the consistency of search strings and the number of records being retrieved after the second pilot, we finalised the work instruction with minor changes to add clarity and transparency. PubReMiner searches were reviewed by the project lead (AD) for quality purposes and to ensure that only terms arising from the word frequency tables produced by PubReMiner were used to develop the search strategy.

### 2.3 Reference Standard

In accordance with best practice, for each review, a complete and comprehensive search of multiple databases, grey literature and reference lists of included studies was undertaken. These searches were used as the reference standard, with the results from each of the conventional Medline and PubReMiner searches compared with the total number of included records arising from the complete and comprehensive search for that review.

This allowed for scenarios where the PubReMiner searches may find included records that were not found by the conventional MEDLINE search; possibly due to the omission of key words or MeSH terms. The exact databases and sources varied according to the research questions and topic area in the given review; further details for each review can be found in Table 1 and on HIQA's Zenodo repository.

**Table 1:** Characteristics of Included Reviews.

Review no.	Brief description	Review type	Review area	Search date*	PMIDs**	Zenodo Link
1	^177^Lu Prostate Specific Membrane Antigen (PSMA) Radioligand Therapy	Review of reviews	Clinical effectiveness	August 2023	21	Link
2	Abdominal Aortic Aneurysm (AAA) Screening	Systematic review	Clinical effectiveness	November 2023	58	Link
3	Bowel Cancer Screening	Systematic review	Cost effectiveness	January 2024	40	Link
4	Bowel Cancer Screening	Systematic review	Test accuracy	March 2024	19	Link
5	Infectious Disease Outbreak Costing Tool	Systematic review	Resource Utilisation Measurement Tools	April 2024	13	Link
6	Magnetic Resonance Guided Radiotherapy (MRgRT)	Systematic review	Clinical effectiveness	May 2024	23	Link
7	Teledermatology	Systematic review	Clinical effectiveness	June 2024	13	Link
8	Bowel Cancer Screening	Systematic review	Clinical effectiveness	July 2024	11	Link
9	Fertility Preservation	Scoping review	International policy and guidelines	July 2024	5	Link
10	Decision‐Making And Oversight of Medical Devices Reimbursement	Scoping review	International policy and guidelines	September 2024	4	Link
11	Respiratory Syncytial Virus (RSV) Immunisation	Systematic review	Clinical effectiveness	September 2024‡	25	Link
12	Teledermatology	Systematic review	Cost effectiveness	November 2024	9	Link

* Date of comprehensive peer‐reviewed search, which included the conventional MEDLINE search. PubReMiner searches generally conducted within 1 calendar week of a conventional search.

** Number of PMIDs provided to PubReMiner to generate the word frequency tables.

^‡^ *PubReMiner search conducted within 12 calendar weeks due to logistical issues and staff leave. PubReMiner searches re‐ran with search limit set up to the 31 August 2024*.

### 2.4 Outcomes

Outcomes were pre‐specified at registration of this SWAR on the SWAR Repository Store; however, definitions were later amended or updated to fit our specific application and investigation [10].

Primary outcomes for each search included:

1. **Sensitivity:** proportion of studies included in the host review that would have been (or were) retrieved by the given MEDLINE search strategy.
$$\frac{\mathrm{MEDLINE}\;\mathrm{Records}\;\mathrm{Retrieved}\&\mathrm{Included}\;\mathrm{in}\;\mathrm{the}\;\mathrm{Review}}{\mathrm{Total}\;\mathrm{Number}\;\mathrm{of}\;\mathrm{Included}\;\mathrm{Studies}\;\mathrm{in}\;\mathrm{the}\;\mathrm{Review}\;(\mathrm{All}\;\mathrm{Searches}\&\mathrm{Sources})}$$
2. **Precision:** proportion of all records retrieved by the given MEDLINE search strategy that were included in the host review.

$$\frac{\mathrm{MEDLINE}\;\mathrm{Records}\;\mathrm{Retrieved}\;\&\;\mathrm{Included}\;\mathrm{in}\;\mathrm{the}\;\mathrm{Review}}{\mathrm{Total}\;\mathrm{Number}\;\mathrm{of}\;\mathrm{MEDLINE}\;\mathrm{Records}\;\mathrm{Retrieved}}$$

Definitions were adapted from the Cochrane Handbook for this SWAR to reflect evaluation of MEDLINE‐only strategies against a reference standard derived from each review's comprehensive multi‐source search. In this SWAR, the review's final set of included studies was treated as the set of relevant records for assessing search retrieval performance, i.e., the reference standard (see Section 2.3). The Cochrane Handbook defines the denominator for sensitivity as “all relevant records in the resource”; however, for this application we operationalised relevance using the host review's final included set, reflecting: (i) recall in the context of multi‐database evidence synthesis, (ii) epistemic uncertainty regarding whether all relevant records were truly identified by any individual database search (particularly in rapid reviews), and (iii) real‐world application of the tool [12].

Secondary outcomes included:

1. **Number needed to read (NNR):** number of references a researcher must screen/read to identify a relevant reference in each search strategy.
2. **Number of unique references:** number of included references retrieved from a database search that were not retrieved from the conventional search.
3. **Efficiency:** time taken by each librarian to construct the search strategy.

### 2.5 Data Management and Analysis

**Figure 1:**
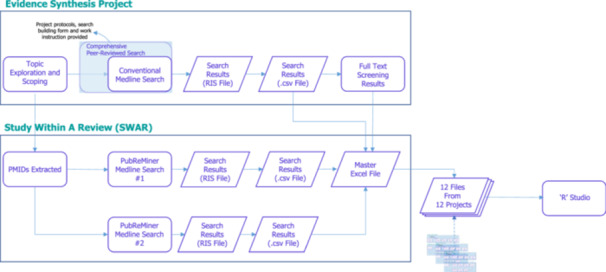
Schematic of the Study Within A Review (SWAR) Design.

For each search, Research Information System (RIS) files were saved and exported to Excel. These were merged into a single Excel file per review, containing all included studies and citations retrieved by the conventional MEDLINE search and each PubReMiner search (Figure 1). Datasets were imported into R Studio version 4.3.2 for analysis.

We used the digital object identifier (DOI) to match the included citations to the citations retrieved by the conventional and PubReMiner searches. For records with missing DOIs, we attempted to match on title; all remaining unmatched records were manually screened thereafter using the author list and title to ensure that no included citation was missed due to identifier discrepancies.

Our protocol specified that we would explore the use of meta‐analytical techniques to evaluate the overall performance of the PubReMiner tool. Having considered the heterogeneous reviews included in this SWAR, we determined it was not appropriate to conduct a quantitative synthesis. Instead, we decided to descriptively summarise and compare outcomes between the conventional search and the PubReMiner searches. We used histograms, generated with ggplot2 (v3.4.4), to visualise outcomes. The data were assessed for normality using Shapiro‐Wilks tests, histograms and Q‐Q plots. Wilcoxon signed‐rank tests with a Holm correction for multiple testing were used for pairwise comparisons of sensitivity and precision between the conventional and each PubReMiner search, as the non‐parametric data were paired (i.e., the searches were all conducted on the same database of records). A Friedman test and a Nemenyi post‐hoc test (where the Friedman test was significant) were applied to compare all three searches simultaneously and to conduct pairwise comparisons while adjusting for multiple testing, respectively. Although McNemar's or Gwets A1C tests is often used in diagnostic test accuracy studies, they were not used here because the total number of irrelevant records missed by a search was unknown, preventing the construction of a 2×2 table of binary outcomes [13, 14, 15]. No formal statistical tests were used to assess time taken, as this outcome was self‐reported by librarians and approximate. The data management, analysis and visualisation were independently second‐checked by another researcher (MB).

## 3 Results

### 3.1 Review Characteristics

Of the 15 reviews conducted during the study period, 12 reviews were recruited into the SWAR. Three reviews were excluded from the SWAR for the following reasons. One was excluded as the search strategy was not developed in‐house, and the review instead updated a search developed by a previous scoping review. A second was excluded as the search had to be conducted on Ovid due to the need to use a specific geographical feature not available on EBSCO. The final review was excluded as a search for guidelines on obesity focused on grey literature searching and did not have any pre‐identified articles or PMIDs.

Seven of the 12 included reviews were clinical effectiveness reviews, two were reviews of cost effectiveness, and the remaining three were reviews to inform the development of health policy related to medical devices, fertility sparing interventions and the costing of infectious disease outbreaks. Review 3 met the inclusion criteria of our SWAR, however as the comprehensive search conducted for the review found no relevant records, the sensitivity and precision outcomes were 0% for all searches and the number needed to read was infinite.

An exploratory analysis was first conducted to check for missing DOIs (see Supporting Materials). Matching was improved in Reviews 2 and 7 by employing secondary matching based on titles after first attempting to match on DOIs. Manual screening did not find any additional matches thereafter. Mean missingness was 4.80%. While missingness was < 10% in 10 of the 12 reviews, in two reviews, the missingness was higher. In Review 2, 16% and 17% of DOIs were missing in the conventional and PubReMiner 1 searches, respectively. In Review 3, 19% of the DOIs were missing in the PubReMiner 2 search.

### 3.2 Primary Outcomes

**Figure 2:**
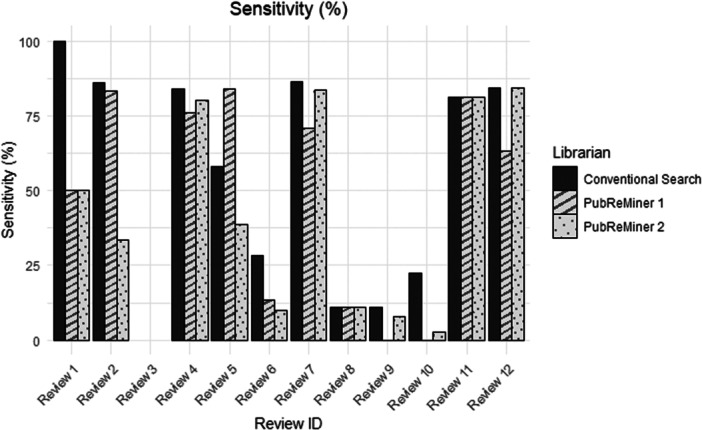
Histogram of sensitivity.

In general, sensitivity outcomes were lower for the PubReMiner searches compared to the conventional MEDLINE search conducted in HIQA (Figure 2). The only exception to this was Review 5, where PubReMiner 1 had a higher sensitivity and found records that were not retrieved by the conventional MEDLINE search (83.87% *vs.* 58.06%, respectively). However, for this review, PubReMiner 2 had a sensitivity that was much lower than that of either of these searches (38.71%). The sensitivity results were generally not consistent between reviews or between the librarians executing the PubReMiner searches. After correcting for multiple testing, the Wilcoxon test showed a statistically significant difference between the conventional MEDLINE search and PubReMiner 2 (*p* = 0.0428), but not between the conventional search and PubReMiner 1 (*p* = 0.194) or between PubReMiner 1 and PubReMiner 2 (*p* = 0.944).

A Friedman test found a statistically significant difference between the three librarians and their searches (*p* = 0.007). Because the Friedman test does not specify which pairs differ, a post‐hoc Nemenyi test was conducted. This found a statistically significant difference between the conventional MEDLINE search and PubReMiner 1 (*p* = 0.028), but not between the conventional search and PubReMiner 2 (*p* = 0.065) or between PubReMiner 1 and PubReMiner 2 (*p* = 0.945).

**Figure 3:**
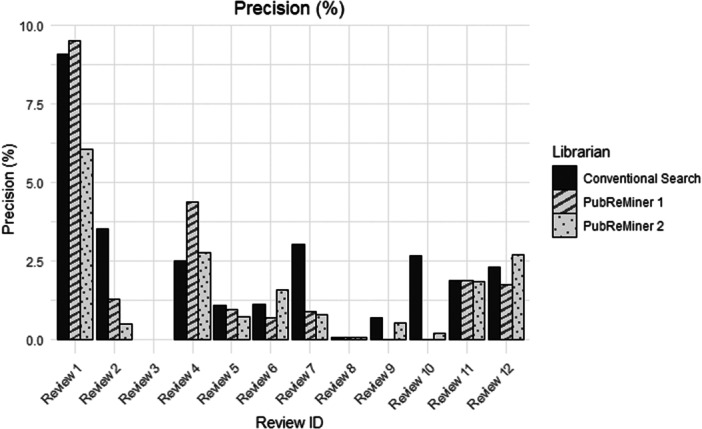
Histogram of precision.

Precision of all searches tended to be low and less than 5%, except for Review 1 (Figure 3). In Review 4, the PubReMiner 1 search produced more precise results than the conventional MEDLINE search (4.38% *vs.* 2.49%), however, this was not replicated by PubReMiner 2 (2.76%). Similarly, in Review 6, PubReMiner 2 had higher precision than the conventional MEDLINE search (1.57% *vs.* 0.69%), the PubReMiner 1 search was lower than the PubReMiner 2 (1.10% *vs.* 1.57%). In other instances, the precision of PubReMiner searches was approximately the same as the conventional search (Reviews 5, 8 and 11), like sensitivity the precision results were not consistent between reviews or between librarians. Wilcoxon signed‐rank tests did not find a statistically significant difference in precision results between the conventional MEDLINE search and PubReMiner 1 (*p* = 0.379) or PubReMiner 2 (*p* = 0.379), nor between PubReMiner 1 and PubReMiner 2 (*p* = 0.638). A Friedman test compared the three searches and did not find a statistically significant difference between these three searches (*p* = 0.183).

### 3.3 Secondary Outcomes

**Figure 4:**
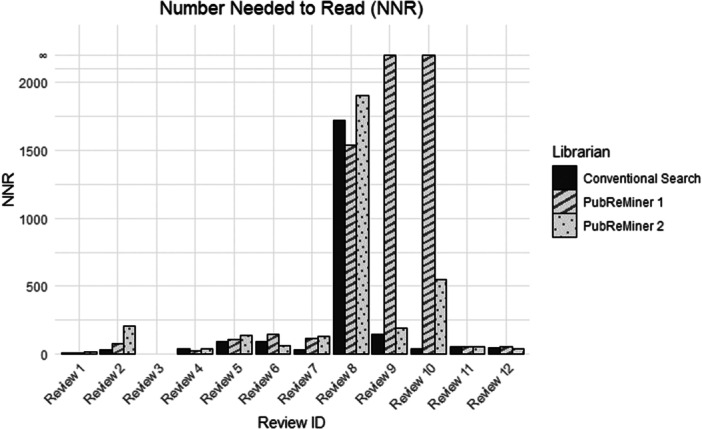
Histogram of number needed to read.

The NNR was lower for the conventional search compared to the PubReMiner searches in Reviews 2, 5, 7, 9 and 10. This was not the case for Reviews 1, 4, 6, 8 and 12 however, in these cases the NNR was lower for one librarian conducting the PubReMiner search and not the other. Wilcoxon signed‐rank tests found no statistically significant difference in NNR results between the conventional MEDLINE search and PubReMiner 1 (*p* = 0.307), the conventional search and PubReMiner 2 (*p* = 0.161) nor between PubReMiner 1 and PubReMiner 2 (p = 1.000). A Friedman test compared the three searches and did not find a statistically significant difference between these three searches (*p* = 0.183). Where the precision was 0% (and the search did not obtain any records included in the final review), the number needed to read approached infinity (Figure 4).

**Figure 5:**
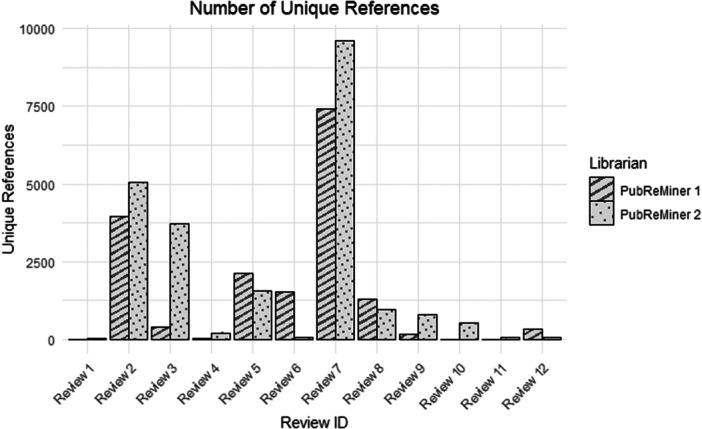
Histogram of number of unique reference.

**Figure 6:**
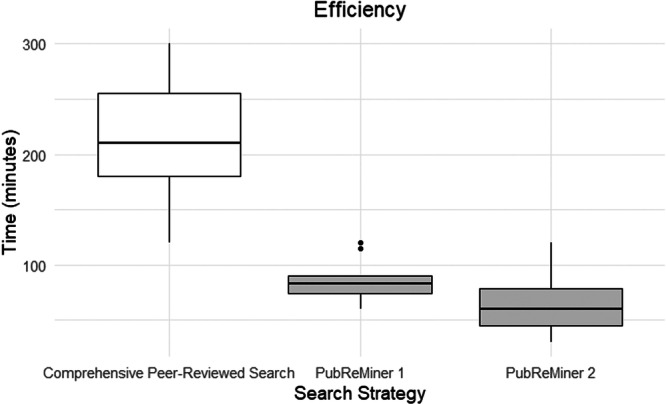
Box plot of efficiency.

The number of unique references is presented in Figure 5 for the PubReMiner 1 and 2 searches. Although there was some variation between PubReMiner 1 and PubReMiner 2 in terms of their unique references, the difference was not statistically significant (*p* = 0.339). Time taken to complete the search was an approximate, self‐reported estimate by librarians. However, boxplots presented in Figure 6 show that in general, the PubReMiner searches were substantially faster than the overall comprehensive search conducted by HIQA.

## 4 Discussion

This SWAR evaluated PubReMiner‐generated searches compared to conventional MEDLINE searches. While statistical significance was only observed in a few comparisons, the results revealed substantial variability across reviews and between librarians. In several cases, PubReMiner searches had slightly higher sensitivity or precision than the conventional MEDLINE search; however, these improvements were inconsistent and did not persist across reviews. Overall, the findings suggest that although PubReMiner‐driven searches can occasionally produce comparable performance, it does not do so reliably enough for routine use. Our results are broadly consistent with a previously reported smaller study of text‐mining tools applied to systematic reviews conducted in a number of institutes; however, in that study, the use of these tools was not prescribed, librarians could choose from a variety of tools, and a smaller number of outcomes were examined [16]. The authors of that study have since published a paper outlining practical considerations and emphasizing that the way these tools are applied, alone or in combination with others, may yield different results [17].

The variability in outcomes between reviews has also been seen elsewhere. One study used a large dataset of 10,346 natural language descriptions of reviews and Boolean searches to train an LLM and evaluated it on a dataset of 57 systematic reviews through semi‐structured interviews with eight librarians [18]. The model‐generated queries demonstrated a sensitivity of 85% and an NNR of 1,206, though these outcomes exhibited markedly wide interquartile ranges (40%–100% and 205–5,810, respectively). Interviewees suggested the models lacked both the necessary sensitivity and precision to be used without scrutiny.

In addition to variability between reviews, important inter‐operator variability was observed between the two librarians conducting PubReMiner searches in this SWAR. This occurred despite significant efforts to develop a step‐by‐step work instruction and two pilot reviews to test the feasibility of our methods. Each librarian had the same set of PMIDs, access to the review protocol and a pre‐defined search‐building form which outlined the key concepts to be included as part of the search. These differences likely stemmed from how each librarian selected and combined keywords and MeSH terms from PubReMiner's frequency tables. Although LLMs have not been promising in search strategy development to date [19], this may be a challenge that future AI tools can address. However, they will first have to overcome the fact that important key words and MeSH terms may not be found in the fields (e.g., title, abstract and MeSH terms) that tools such as PubReMiner use to generate their frequency tables. Access to the full PDF versions of these articles (in PDF readable formats) might improve their ability to generate sensitive and precise searches. However, such tools may also have to overcome the paywall barriers that exist.

While acknowledging the limitations of human‐led approaches (where people drive the core decisions, interpretation, and methodological design of the evidence‐synthesis process but AI may be used as a tool) and AI‐led approaches (where AI performs major components of the evidence synthesis workflow with minimal human intervention), there is a question as to how well these approaches could be combined to achieve better results. Spillias et al. previously concluded that while generative AI struggled with search string development, AI‐human collaboration outperforms both AI and librarians working on their own [20]. This study by Spillias et al. did not assess outcomes such as sensitivity and precision, and there is a general lack of large and validated evaluations supporting the use of such tools, as highlighted by the *Cochrane 2024 Handbook* [12]. A previous case study of text‐mining methods by O'Keefe et al. demonstrated the potential of such tools as a complement to traditional methods; the reliance on five preselected seed articles and the niche topic of the case study may limit the generalisability of its findings [21]. It is possible that generative AI and tools such as PubReMiner have a complementary role in addition to current best practice, without proven improvements in sensitivity and precision, such tools may inadvertently reduce efficiency if they are adding additional steps to the overall strategy development process.

Our study has two important strengths and contributions to note. Firstly, the overall aim of our SWAR closely aligns with the Priority III study, a series of surveys and workshops involving various stakeholders, which identified research priorities for improving how we plan, do and share the results of rapid reviews in the context of healthcare [22]. In particular, Priority III raised important questions about the best approaches for developing search strategies for rapid reviews, and our study indicates that the PubReMiner approach may offer potential time‐saving benefits. However, while we acknowledge that readers may have different views on what constitutes ‘acceptable sensitivity’, we do not recommend that our approach of using PubReMiner for search strategy building be used, based on the results observed in this SWAR. Equally, the findings demonstrate that researchers should look for transparent evaluations of similar statistical or AI tools used in evidence synthesis before employing them in their routine research practices.

Secondly, with respect to our study design, it has been recognised that there is a lack of SWARs that are embedded across multiple reviews to answer methodological research questions [1]. The multi‐review SWAR approach taken here has a number of distinct advantages over more cross‐sectional meta‐epidemiological or research‐on‐research studies. Firstly, it allows the investigator to better control for temporality, as search filters are subject to error when it comes to filtering by precise dates. Hence, a traditional methodological study, which takes a number of systematic reviews and re‐runs of searches, may find references published after the original search was conducted. There may also be important differences between these reviews in how authors conducted their searches and what the novel search strategy is compared to, whereas with our SWAR, we were able to follow pre‐defined work instructions for the novel method and the comparator each and every time.

A number of limitations in our study are worth highlighting. First of all, although we exceeded the recommended minimum sample size of five reviews for using a Wilcoxon signed rank test, the only statistically significant difference found was with respect to sensitivity. Our sample may have lacked power to detect smaller, yet potentially meaningful, differences in other outcomes, such as precision, which was < 5% in most searches and reviews. Secondly, although unique references were found in the PubReMiner searches, we decided not to screen these references for eligible records that may have been missed by the comprehensive search, as SWARs and Studies Within Trials (SWATs) are designed to not to interfere with their host review or trials. Finally, although the self‐reported time taken was relatively short for the PubReMiner searches, this could only be compared to the comprehensive search and not just the portion of time spent on the conventional MEDLINE search. Hence, efficiency results should be interpreted with caution.

This study illustrates that a SWAR embedded in multiple reviews can answer methodological research questions that a SWAR embedded in only one review cannot. While multi‐review SWARs may be limited to a select number of settings where there is a high volume of evidence synthesis outputs, it is hoped that this multi‐review SWAR approach may be of interest to other methodological researchers so that they can also answer important methodological questions that cannot be answered within a single review.

## 5 Conclusion

We found that although PubReMiner‐generated search strategies may offer notable time‐saving benefits, their sensitivity and precision were inconsistent and generally inferior to conventional methods. Furthermore, substantial variability was observed both between reviews and among users of the tool. This study illustrates the value of multi‐review SWARs in producing more generalisable and practice‐relevant methodological insights.

## Author Contributions

**Andrew Dullea:** conceptualization, methodology, data curation, validation, formal analysis, funding acquisition, visualization, project administration, writing – original draft, writing – review and editing and investigation. **Marie Carrigan:** conceptualization, methodology, investigation, writing – review and editing and project administration. **Lydia O'Sullivan:** conceptualization, methodology, supervision and writing – review and editing. **Isabelle Delaunois:** methodology, writing – review and editing and investigation. **Helen Clark:** methodology, investigation and writing – review and editing. **Martin Boudou:** methodology, writing – review and editing and validation. **Martina Giusti:** methodology and writing – review and editing. **Kieran A Walsh:** methodology, writing – review and editing and supervision. **Patricia Harrington:** methodology and writing – review and editing. **Susan M Smith:** methodology, writing – review and editing and supervision. **Máirín Ryan:** writing – review and editing and funding acquisition.

## Conflicts of Interest

The authors declare that they have no known competing financial interests or personal relationships that could have appeared to influence the work reported in this paper.

## SWAR Store Registration

SWAR 26.

## Supporting information

CESM SWAR SuppMaterials.

## Data Availability

The data that support the findings of this study are openly available from Zenodo: https://zenodo.org/communities/hiqa-repository/records?q=&l=list&p=1&s=10&sort=newest. Further data may be made available upon request.

## References

[1] D. Devane , N. N. Burke , S. Treweek , et al., “Study Within a Review (SWAR),” Journal of Evidence‐Based Medicine 15, no. 4 (2022): 328–332, 10.1111/jebm.12505. 36513956 PMC10107874

[2] I. Shemilt , D. Caldwell , D. Edwards , M. Halicka , S. Harnan , and M. Clarke The SWARs Living Map: A Living Map of Studies Within Reviews (SWARs) (2025), https://eppi.ioe.ac.uk/eppi-vis/Review/Index/745.

[3] ResearchRabbit. ResearchRabbit: Reimagine Research. 2025, https://www.researchrabbit.ai/.

[4] Semantic Scholar. Semantic Scholar: A free, AI‐powered Research Tool for Scientific Literature. 2025, https://www.semanticscholar.org/.

[5] Amsterdam University Medical Center. PubMed PubReMiner. 2025, https://hgserver2.amc.nl/cgi-bin/miner/miner2.cgi.

[6] OpenAI. ChatGPT. 2025, https://chatgpt.com/.

[7] E. E. Johnson , H. O'Keefe , A. Sutton , and C. Marshall , “The Systematic Review Toolbox: Keeping Up to Date With Tools to Support Evidence Synthesis,” Systematic Reviews 11, no. 1 (December 2022): 258, 10.1186/s13643-022-02122-z. 36457048 PMC9713957

[8] E. Hausner , C. Guddat , T. Hermanns , U. Lampert , and S. Waffenschmidt , “Development of Search Strategies for Systematic Reviews: Validation Showed the Noninferiority of the Objective Approach,” Journal of Clinical Epidemiology 68, no. 2 (February 2015): 191–199, 10.1016/j.jclinepi.2014.09.016. 25464826

[9] L. Slater , “Pubmed Pubreminer,” Journal of the Canadian Health Libraries Association/Journal de l'Association des bibliothèques de la santé du Canada 33, no. 2 (2014): 106–107, 10.5596/c2012-014.

[10] A. Dullea , M. Carrigan , S. M. Smith , et al. SWAR 26: Sensitivity and Completeness of Search Strategies Built Using a Text‐mining Word Frequency Tool (PubReMiner) Compared to Current best Practice for Building a Search Strategy. SWAR Repository Store, accessed Ongoing, https://www.qub.ac.uk/sites/TheNorthernIrelandNetworkforTrialsMethodologyResearch/FileStore/SWARFileStore/SWAR26%20A%20Dullea,%20M%20Carrigan,%20S%20Smith,%20L%20O%E2%80%99Sullivan,%20M%20Giusti,%20I%20Delaunois,%20H%20Clark,%20K%20Walsh,%20P%20Harrington,%20M%20Ryan%20(2023%20MAR%201%202010).pdf. 10.1002/cesm.70074 PMC12915468 41716537

[11] J. McGowan , M. Sampson , D. M. Salzwedel , E. Cogo , V. Foerster , and C. Lefebvre , “PRess Peer Review of Electronic Search Strategies: 2015 Guideline Statement,” Journal of Clinical Epidemiology 75 (July 2016): 40–46, 10.1016/j.jclinepi.2016.01.021. 27005575

[12] C. Lefebvre , J. Glanville , S. Briscoe , et al. Chapter 4: Searching for and Selecting Studies. [Last Updated March 2025]. In: Higgins JPT, Thomas J, Chandler J, Cumpston M, Li T PM, VA W, eds. *Cochrane Handbook for Systematic Reviews of Interventions Version 651*. Cochrane, 2025; 2024.

[13] S. Kim and W. Lee , “Does McNemar's Test Compare the Sensitivities and Specificities of Two Diagnostic Tests?,” Statistical Methods in Medical Research 26, no. 1 (February 2017): 142–154, 10.1177/0962280214541852. 24996898

[14] A. Trajman and R. R. Luiz , “McNemar χ2 Test Revisited: Comparing Sensitivity and Specificity of Diagnostic Examinations,” Scandinavian Journal of Clinical and Laboratory Investigation 68, no. 1 (January 2008): 77–80, 10.1080/00365510701666031. 18224558

[15] N. Wongpakaran , T. Wongpakaran , D. Wedding , and K. L. Gwet , “A Comparison of Cohen's Kappa and Gwet's AC1 When Calculating Inter‐Rater Reliability Coefficients: A Study Conducted With Personality Disorder Samples,” BMC Medical Research Methodology 13, no. 1 (April 2013): 61, 10.1186/1471-2288-13-61. 23627889 PMC3643869

[16] R. A. Paynter , R. Featherstone , E. Stoeger , C. Fiordalisi , C. Voisin , and G. P. Adam , “A Prospective Comparison of Evidence Synthesis Search Strategies Developed With and Without Text‐Mining Tools,” Journal of Clinical Epidemiology 139 (November 2021): 350–360, 10.1016/j.jclinepi.2021.03.013. 33753230

[17] G. P. Adam and R. Paynter , “Development of Literature Search Strategies for Evidence Syntheses: Pros and Cons of Incorporating Text Mining Tools and Objective Approaches,” BMJ Evidence‐Based Medicine 28, no. 2 (2023): 137–139, 10.1136/bmjebm-2021-111892. 35346974

[18] G. P. Adam , J. DeYoung , A. Paul , et al., “Literature Search Sandbox: A Large Language Model That Generates Search Queries for Systematic Reviews,” JAMIA Open 7, no. 3 (Oct 2024): ooae098, 10.1093/jamiaopen/ooae098. 39323560 PMC11424077

[19] J.‐L. Lieberum , M. Töws , M.‐I. Metzendorf , et al., “Large Language Models for Conducting Systematic Reviews: on the Rise, but Not yet Ready for Use – a Scoping Review,” Journal of Clinical Epidemiology 181 (May 2024): 111746, 10.1016/j.jclinepi.2025.111746. 40021099

[20] S. Spillias , P. Tuohy , M. Andreotta , et al., “Human‐Ai Collaboration to Identify Literature for Evidence Synthesis,” Cell Reports Sustainability 1, no. 7 (2024): 100132, 10.1016/j.crsus.2024.100132.

[21] H. O'Keefe , J. Rankin , S. A. Wallace , and F. Beyer , “Investigation of Text‐Mining Methodologies to Aid the Construction of Search Strategies in Systematic Reviews of Diagnostic Test Accuracy‐A Case Study,” Research Synthesis Methods 14, no. 1 (January 2023): 79–98, 10.1002/jrsm.1593. 35841125 PMC10088010

[22] C. Beecher , E. Toomey , B. Maeso , et al., “Priority III: Top 10 Rapid Review Methodology Research Priorities Identified Using a James Lind Alliance Priority Setting Partnership,” Journal of Clinical Epidemiology 151 (November 2022): 151–160, 10.1016/j.jclinepi.2022.08.002. 36038041 PMC9487890

