# Supplementary material for: Sensitivity and Precision of Search Strategies Built Using a Text‐Mining Word Frequency Tool (PubReMiner) Compared to Current Best Practice for Building Search Strategies: A Study Within a Review (SWAR)
Source: Cochrane Evid Synth Methods. 2026 Feb 18;4(2):e70074. doi: 10.1002/cesm.70074 (PMC12915468; doi:10.1002/cesm.70074)
Supplement: Supplementary file 1 — CESM SWAR SuppMaterials. [file CESM-4-e70074-s001.docx]

# Supplementary Material

Table 2: The sensitivity of searches for each review

|  | Conventional Search | PubReMiner 1 | PubReMiner 2 |
| --- | --- | --- | --- |
| Review 1 | 100.00 | 50.00 | 50.00 |
| Review 2 | 85.90 | 83.33 | 33.33 |
| Review 3 | NA | NA | NA |
| Review 4 | 84.00 | 76.00 | 80.00 |
| Review 5 | 58.06 | 83.87 | 38.71 |
| Review 6 | 28.37 | 13.48 | 9.93 |
| Review 7 | 86.36 | 70.91 | 83.64 |
| Review 8 | 11.11 | 11.11 | 11.11 |
| Review 9 | 11.11 | 0.00 | 7.94 |
| Review 10 | 22.22 | 2.78 | 2.78 |
| Review 11 | 81.25 | 81.25 | 81.25 |
| Review 12 | 84.21 | 63.16 | 84.21 |

Table 3: The precision of searches for each review

|  | Conventional Search | PubReMiner 1 | PubReMiner 2 |
| --- | --- | --- | --- |
| Review 1 | 9.09 | 9.52 | 6.06 |
| Review 2 | 3.51 | 1.28 | 0.48 |
| Review 3 | NA | NA | NA |
| Review 4 | 2.49 | 4.38 | 2.76 |
| Review 5 | 1.06 | 0.95 | 0.71 |
| Review 6 | 1.10 | 0.69 | 1.57 |
| Review 7 | 3.03 | 0.87 | 0.77 |
| Review 8 | 0.06 | 0.07 | 0.05 |
| Review 9 | 0.68 | 0.00 | 0.52 |
| Review 10 | 2.65 | 0.00 | 0.18 |
| Review 11 | 1.88 | 1.88 | 1.83 |
| Review 12 | 2.31 | 1.74 | 2.69 |

Table 4: The number needed to read (NNR) for each review

|  | Conventional Search | PubReMiner 1 | PubReMiner 2 |
| --- | --- | --- | --- |
| Review 1 | 11.00 | 10.50 | 16.50 |
| Review 2 | 28.48 | 78.05 | 206.69 |
| Review 3 | NA | NA | NA |
| Review 4 | 40.24 | 22.84 | 36.20 |
| Review 5 | 94.06 | 104.96 | 141.42 |
| Review 6 | 91.12 | 145.79 | 63.57 |
| Review 7 | 33.04 | 114.99 | 129.78 |
| Review 8 | 1723.00 | 1535.50 | 1907.00 |
| Review 9 | 147.71 | ∞ | 193.60 |
| Review 10 | 37.75 | ∞ | 550.00 |
| Review 11 | 53.15 | 53.15 | 54.54 |
| Review 12 | 43.25 | 57.58 | 37.13 |

Table 5: The number of unique references for each review

|  | PubReMiner 1 | PubReMiner 2 |
| --- | --- | --- |
| Review 1 | 6 | 19 |
| Review 2 | 3966 | 5037 |
| Review 3 | 390 | 3724 |
| Review 4 | 13 | 207 |
| Review 5 | 2111 | 1553 |
| Review 6 | 1525 | 68 |
| Review 7 | 7407 | 9628 |
| Review 8 | 1273 | 946 |
| Review 9 | 150 | 781 |
| Review 10 | 0 | 538 |
| Review 11 | 6 | 51 |
| Review 12 | 315 | 68 |

Figure 7: Missing Digital Object Identifier (DOI) Histogram


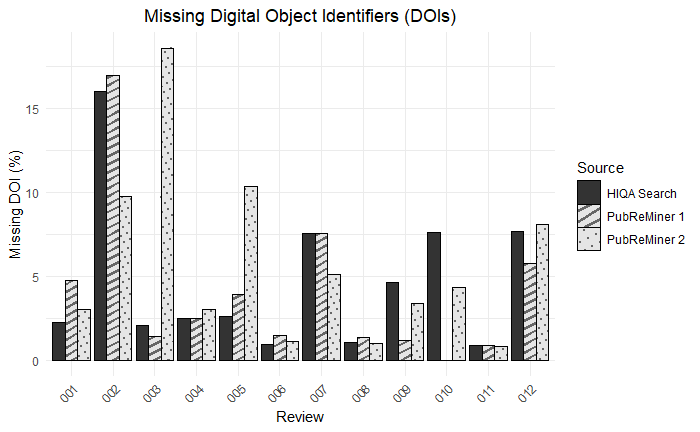


## Conventional Work Instruction

| GETTING STARTED | |
| --- | --- |
| 1.1 | - You will receive a draft protocol for the review project. This document outlines the objectives for the project and sets out the methods to be used. The protocol includes the research question(s) and PICOS and exclusion and inclusion criteria. - In your search planning form^^[[1]](#footnote-1)^^, divide the question into key concepts and select the concepts to include in the literature search. - Together with the Principal Investigator (PI), you will need to inform the PubReMiner librarian of the key concepts and filters to be used. |
| 1.2 | **NOTE:** It is at this point you should start the timer to record the total time taken to carry out this work.   - Identify relevant MeSH and/or other thesauri as appropriate. - Note index terms on the search planning form. - Add variations in search terms: truncation; spelling variants; abbreviations; opposites.^^[[2]](#footnote-2)^^ - Document your search strategy on the search planning form^1^ as you progress through work instruction. For each concept, write down the relevant MeSH terms/free text terms including wildcards, proximity and phrase searching. |
| SEARCHING MEDLINE | |
| 2.1 | - Run the search in Medline via EBSCOhost. For keywords restrict to TITLE or ABSTRACT. If you wish to include a search for substances, search in ALL FIELDS. - If the search restricts to a particular study design e.g. systematic review, RCT etc. use an appropriate study design filter. This must be communicated to the librarian running the PubReMiner search strategy to ensure the results are comparable. - Optimise the search.^[[3]](#footnote-3)^ - Apply a date limit and any other limits as agreed with the requestor.   **NOTE:** It is at this point you should stop the timer to record the total time taken to carry out this work.   - Ask a HSE Evidence Team Librarian to peer review your draft search strategy using Peer-Review of Electronic Search Strategies (PRESS) checklist - Save the search in EBSCOhost. Name it: Conventional_project name |
| MANAGING REFERENCES | |
| 3.1 | - Save results as an RIS file. You will need to forward these to the P.I. at the end ([adullea@hiqa.ie](mailto:adullea@hiqa.ie)). - The P.I. will save as an Endnote library and export the library as an excel file, which will then be uploaded to SharePoint. |
| DOCUMENTING THE SEARCH | |
| 4.1 | - Create a new word document entitled “Conventional_project name” Cut and paste the search strategy used in Medline. - You should also note the time taken to conduct this work at the end of this document. - Two files should be sent to the P.I. in a single email via [adullea@hiqa.ie](mailto:adullea@hiqa.ie).  1. An RIS file with the search results. 2. A word document with the search string used in Medline (EBSCO) & total time taken to conduct the work  - The P.I. will rename and save the files in SharePoint location. |

## PubReMiner Work Instruction

| Selecting PMIDs | |
| --- | --- |
| 1.1 | The Principal Investigator (P.I.) will review papers sent in by undertakings to support their applications, and shall review the initial topic exploration conducted by the project lead (or lead researcher). PMIDs will be chosen if they are relevant to the research question(s) asked, and will be distinguished from other records which simply provide background or contextual information, or do not fully relate to the research question(s). |
| GETTING STARTED | |
| 2.1 | - You will receive a draft protocol for the review project. This document outlines the objectives for the project and sets out the methods to be used. The protocol includes the research question(s) and PICOS and exclusion and inclusion criteria. - Familiarise yourself with the research project as outlined in the protocol and make a note of possible key phrases and keywords. |
| 2.2 | - The P.I. together with HIQA’s librarian carrying out the conventional search strategy will meet with the librarian running the PubReMiner search and outline the key concepts for the search strategy. - If a study design filter is to be used, this will be communicated as will other limits to be applied to the search e.g. date limits, restrictions by language etc. |
| SEARCHING PUBREMINER | |
| 3.1 | - 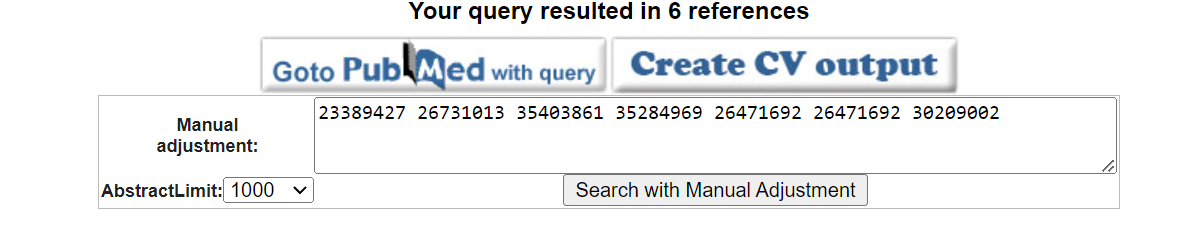The P.I. will send PMIDs for relevant literature discovered during topic exploration. A minimum of 4 PMIDS will be provided. - Input all PMIDs into PubReminer: <https://hgserver2.amc.nl/cgi-bin/miner/miner2.cgi>   **NOTE:** It is at this point you should start the timer to record the total time taken to carry out this work. |
| 3.2 | PubReMiner is a text and frequency analysis tool that works with Pubmed data to generate a list of high frequency words and MeSH terms.   - If you’re unsure about the appropriateness of a certain MeSH term you can search for it in Pubmed’s MeSH database: <https://www.ncbi.nlm.nih.gov/mesh/> and read the scope note for clarification. - If you’re unsure about the meaning of a free text term, you could check for a definition in Medline plus <https://medlineplus.gov/encyclopedia.html> or a similar resource. - PubReMiner automatically merges similar words together and displays the smallest form of the word. Saving your PMID search as a txt file will show you the full list of alternative words.   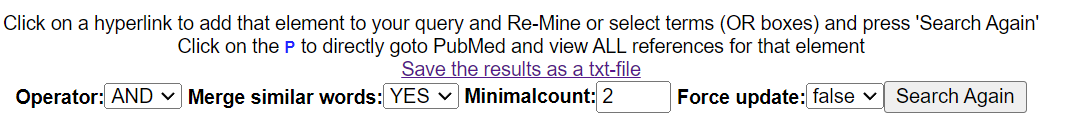   - For free text text terms, you can use wildcard and truncation symbols where appropriate to create searches with unknown characters, multiple spellings or various endings. Instead of using proximity operators, combine two words that are commonly seen together using the boolean operator “AND” or search for two words together as a phrase.   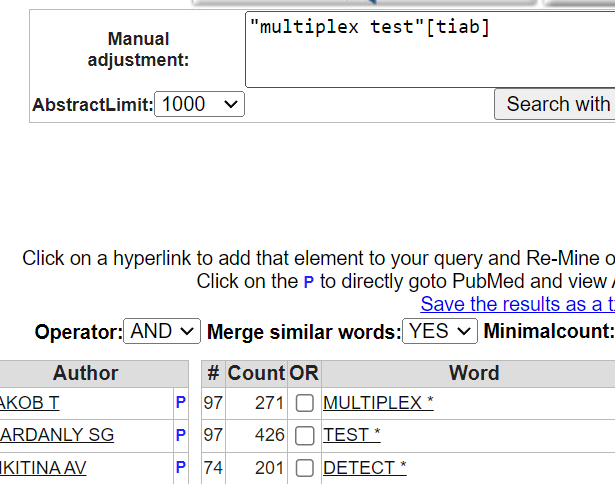  It makes more sense to search for the phrase: “multiplex* test*” instead of searching multiplex* OR test*  Alternatively you could combine two words with the boolean operator “AND” e.g. multiplex* AND test*   - In Pubreminer, MeSH terms are often displayed with subheadings e.g. Neuroendocrine Tumors/metabolism. You can remove the subheading and explode the MeSH term if appropriate e.g. MH "Neuroendocrine Tumors+"You can only search for words or MeSH terms that appear on the list generated by PubReMiner. - Terms and phrases may not be taken from the protocol or other sources, only those generated by the PubReMiner tool may be used. - If you are uncertain as to whether to include a PubReMiner term or not, you may consult with the project lead within the ionising radiation team for advice. You may not ask for additional terms or key words from the project lead. - You do not have to use all the words and MeSH terms generated by PubReMiner, you can select the terms that you think are appropriate for each concept of your search strategy. - Only MeSH terms that are equivalent to the free text terms should be used to keep the search as precise as possible. For example, for the ‘EOS system’ there is no equivalent MESH term, ‘3D imaging’ is too broad a MeSH term to be used. The protocol may assist you in exercising judgement in this regard. - Results from PubReMiner should be saved as a txt-file as shown above. ‘Merge similar words’ should be left as a ‘YES’ when saving. These will need to be forwarded to [adullea@hiqa.ie](mailto:adullea@hiqa.ie) at the end. |
| 3.3 | - For each concept, write down the relevant MeSH terms/free text terms in your search planning form^1^ including wildcards, proximity and phrase searching. |
| SEARCHING MEDLINE | |
| 4.1 | - Run the search in Medline via EBSCOhost. For keywords restrict to TITLE or ABSTRACT. If you wish to include a search for substances, search in ALL FIELDS. - If the search restricts to a particular study design e.g. systematic review, RCT etc. use an appropriate study design filter as agreed with the librarian running the “conventional search strategy”. - The number of results retrieved should be considered reasonable. If the research question is focused and the search strategy appears too imprecise and retrieves a significant number of results (e.g. >10,000) consider removing free text terms/MeSH terms that are very broad and do not directly relate to the research question. Care should be taken to ensure relevant results are not removed in the process of trying to optimise the search. |
| 4.2 | - Save the search in EBSCOhost. Name it: PubReminer_project name   **NOTE:** It is at this point you should stop the timer to record the total time taken to carry out this work. The date of the search should also be saved. |
| MANAGING REFERENCES | |
| 5.1 | - Save results as an RIS file. You will need to forward these to the P.I. at the end ([adullea@hiqa.ie](mailto:adullea@hiqa.ie)). - The P.I. will save as an Endnote library and export the library as an excel file, which will then be uploaded to SharePoint. |
| DOCUMENTING THE SEARCH | |
| 6.1 | - Create a new word document entitled “PubReMiner_project name” Cut and paste the search strategy used in Medline. - You should also note the time taken to conduct this work at the end of this document.   Three files should be sent to the P.I. in a single email via [adullea@hiqa.ie](mailto:adullea@hiqa.ie).   1. Text file with the data mining results from PubReMiner 2. An RIS file with the search results. 3. A word document with the search string used in Medline (EBSCO), date search was run & total time taken to conduct the work  - The P.I. will rename and save the files in SharePoint location. |

## Search Planning Form

|  | Concept 1 | Concept 2 | Concept 3 | Concept 4 |
| --- | --- | --- | --- | --- |
| Key concepts |  |  |  |  |
| **Free text terms** |  |  |  |  |
| MeSH terms |  |  |  |  |

1. See Appendix A: Search Planning Form [↑](#footnote-ref-1)
2. Bramer: do not truncate a word stem that is too short. Also, limitations of interfaces should be noted, especially in PubMed where the number of search term variations that can be retrieved by truncation is limited to 600. Both British and American English spelling variants should be searched as free-text keywords in title or abstract. When searching for abbreviations combine with an important word that is relevant to its meaning or use the Boolean NOT to exclude frequently observed, clearly irrelevant results. It is also important to search for the opposites of search terms to avoid bias. [↑](#footnote-ref-2)
3. Bramer: in order to ensure that most or all potentially relevant articles are retrieved, firstly broaden the initial search strategy, increasing the sensitivity of the search. Identify additional search terms by scanning the top retrieved articles sorted by relevance and generating additional synonyms. Lastly, extra synonyms may be found in articles that have been assigned index terms but do not register synonyms in title or abstract. Searching for [index terms] not [free-text keywords] will identify missed free-text keywords in title or abstract. Searching for [free text keywords] NOT [index terms] will help identify missed index terms. [↑](#footnote-ref-3)
